# *In Vitro* Membrane Remodeling by ESCRT is Regulated by Negative Feedback from Membrane Tension

**DOI:** 10.1016/j.isci.2019.04.021

**Published:** 2019-04-20

**Authors:** Andrew Booth, Christopher J. Marklew, Barbara Ciani, Paul A. Beales

**Affiliations:** 1 School of Chemistry and Astbury Centre for Structural Molecular Biology, University of Leeds, Leeds LS2 9JT, UK; 2 Department of Chemistry and Centre for Membrane Interactions and Dynamics, University of Sheffield, Sheffield S3 7HF, UK

**Keywords:** Biochemistry, Bioengineering, Cell Biology, Biophysics

## Abstract

Artificial cells can shed new light on the molecular basis for life and hold potential for new chemical technologies. Inspired by how nature dynamically regulates its membrane compartments, we aim to repurpose the endosomal sorting complex required for transport (ESCRT) to generate complex membrane architectures as suitable scaffolds for artificial cells. Purified ESCRT-III components perform topological transformations on giant unilamellar vesicles to create complex “vesicles-within-a-vesicle” architectures resembling the compartmentalization in eukaryotic cells. Thus far, the proposed mechanisms for this activity are based on how assembly and disassembly of ESCRT-III on the membrane drives deformation. Here we demonstrate the existence of a negative feedback mechanism from membrane mechanics that regulates ESCRT-III remodeling activity. Intraluminal vesicle (ILV) formation removes excess membrane area, increasing tension, which in turn suppresses downstream ILV formation. This mechanism for *in vitro* regulation of ESCRT-III activity may also have important implications for its *in vivo* functions.

## Highlights

• ESCRT proteins are used to create compartmentalized artificial cell architectures
• *In vitro* ESCRT activity is weakly dependent on the stoichiometry of Vps20 or Vps24
• ESCRT function is strongly regulated by membrane tension
• Membrane tension provides a negative feedback mechanism to attenuate remodeling

## Graphical Abstract

**Figure:**
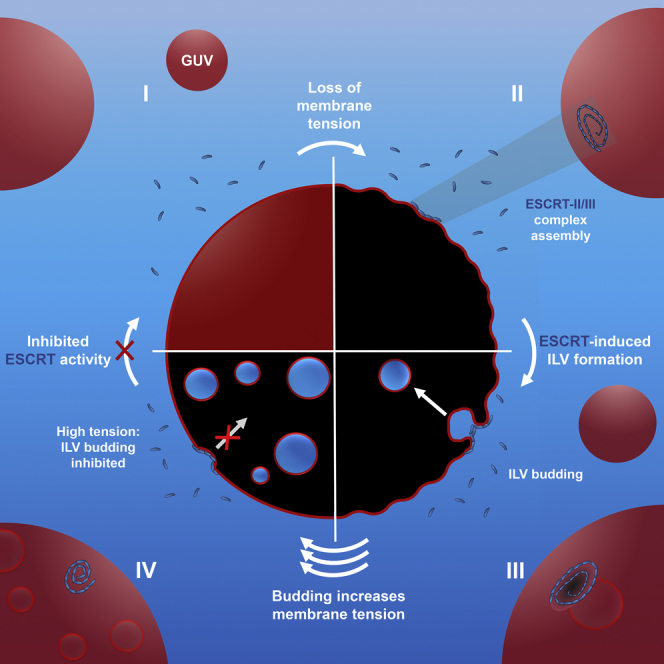

## Introduction

The biological cell is fundamentally a highly complex chemical reactor, where compartmentalization of its chemical processes is essential for complex functions (Ausländer et al., 2017). Eukaryotic organisms contain membrane-bound cellular subcompartments, which allow distinct chemical environments to exist within the cell, such that otherwise incompatible chemistries can be maintained and utilized, e.g., ATP synthesis in the mitochondria and protein degradation in lysosomes (Satori et al., 2013). The manifold capabilities of a cell in the synthesis of complex biomolecules and sensing and response to its environment are all desirable functionalities to emulate within a synthetic system (Chen and Silver, 2012). Therefore chemical technologies that provide control over the formation of multicompartment membrane architectures are fundamental to artificial cell engineering (Buddingh’ and van Hest, 2017, Xu et al., 2016).

Although microfluidic technologies have been developed that allow fixed multicompartment membrane architecture to be formed (Deshpande and Dekker, 2018, Elani et al., 2018), natural cells can dynamically change their compartmentalized architectures. To emulate this dynamic nature within an artificial cell system, we take inspiration from biology by repurposing protein complexes *in vitro* that natively remodel membrane structures (Beales et al., 2015). The “vesicles-within-a-vesicle” architecture we aspire to recreate in an artificial cell bears resemblance to a cellular membrane-bound organelle, the multivesicular body (MVB). In eukaryotic cells, MVBs are formed by the encapsulation of biomolecular cargo into intraluminal vesicles (ILVs) within endosomes (Hanson and Cashikar, 2012). This membrane remodeling event is performed by the ESCRT (endosomal complex required for transport) machinery (Hurley, 2008). This activity lends itself to the possibility of using ESCRT in the construction of multicompartment artificial cells *in vitro* (Marklew et al., 2018).

**Figure 1:**
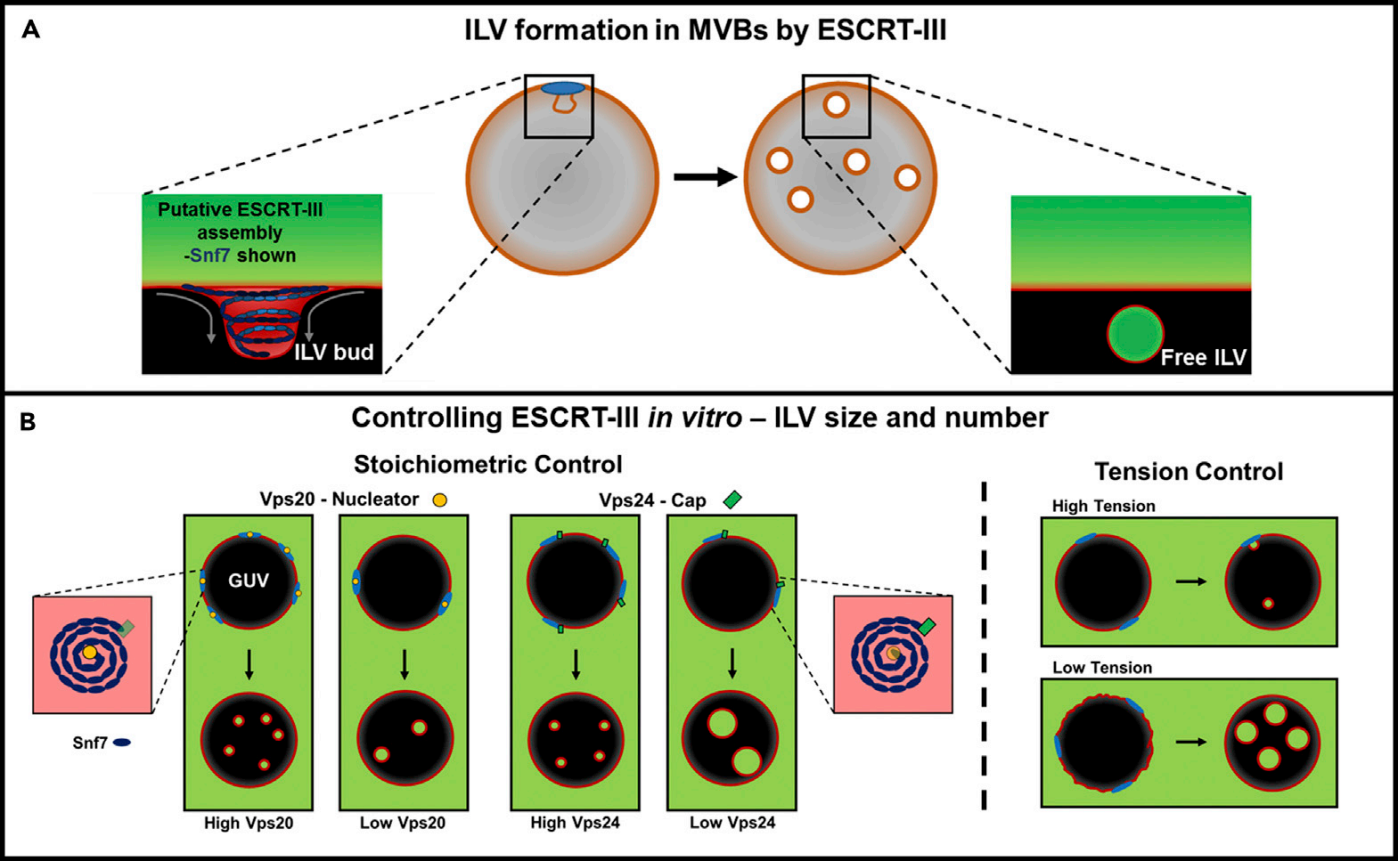
Experimental Design Rationale (A) ESCRT-III drives a number of cellular processes that generate new membrane-bound compartments, notably multivesicular body (MVB) genesis; this process that can be reconstituted *in vitro* using synthetic vesicles (Wollert et al., 2009). (B) ESCRT-III may deform membranes by assembling into ordered supramolecular “springs” (depicted as blue spirals). Protein stoichiometry and membrane tension were identified as variables that may allow finer control over the size and number of intraluminal vesicles (ILVs) produced *in vitro*. Nucleation of Snf7 spiral filaments by Vps20 may provide a means to control the number of ILVs, whereas Vps24 may act as a terminator or “cap” of Snf7 filament growth, limiting ILV size. High membrane tension may impede ILV formation by resisting membrane deformation, potentially affecting both the size and number of ILVs formed.

Human ESCRT drives multiple key membrane-remodeling processes, such as MVB formation, cell division, exosome formation, HIV budding, and plasma and nuclear envelope membrane repair (Christ et al., 2017, Hurley, 2015). In yeast, ESCRT is responsible for cargo sorting in the endosomal pathway and also for nuclear pore complex quality control (Thaller and Lusk, 2018). The ESCRT machinery responsible for transmembrane receptor sorting via the endosomal pathway is composed of ESCRT-0, ESCRT-I, and ESCRT-II protein complexes, which capture ubiquitinated transmembrane cargo at the late endosome membrane and initiate membrane invagination (Frankel and Audhya, 2018). The central membrane remodeling complex is ESCRT-III, a highly conserved set of proteins in eukaryotes, and the AAA + ATPase Vps4 (Figure 1A) (Adell et al., 2016, Monroe and Hill, 2016). In *Saccharomyces cerevisiae*, ESCRT-III consists of a core machinery composed of the Vps20, Snf7, Vps24, and Vps2 subunits. ESCRT-III-mediated membrane budding and scission of vesicles occurs with a unique topology, whereby the membrane is pushed away from the cytosol (Schöneberg et al., 2017). The initial membrane deformation performed by ESCRT-II recruits ESCRT-III subunits and drives the formation of ESCRT-III filaments on the membrane (Henne et al., 2013). *In vitro* studies on flat surface-supported membranes have shown that ESCRT-III forms spirals that are proposed to behave like springs, storing elastic energy, which deform the membrane upon release (Chiaruttini et al., 2015). The observation of this spiral structure informs the mechanical rationale for membrane deformation and scission in many of the proposed mechanisms where either a spiral (Chiaruttini and Roux, 2017), cone (Agudo-Canalejo and Lipowsky, 2018), or dome (Fabrikant et al., 2009, Różycki et al., 2012) structure made by ESCRT-III stabilizes and subsequently drives scission of the ILV bud neck. This process is made possible by the action of the ATPase Vps4, which has been shown to catalyze ESCRT-III activity by maintaining the dynamics of complex assembly and disassembly (Mierzwa et al., 2017, Adell et al., 2017, Schöneberg et al., 2018).

ESCRT-II recruits the ESCRT-III subunit Vps20 to form a complex with affinity for regions of membrane curvature (Fyfe et al., 2011). In turn, Vps20 nucleates the formation of Snf7 spirals that are capped at the growing end by a Vps24-Vps2 complex (Teis et al., 2008). Vps2 recruits the ATPase Vps4 through the interaction between its MIM (MIT-interacting motif) and the MIT (microtubule-interacting and transport) domain of Vps4 (Obita et al., 2007, Stuchell-Brereton et al., 2007). ILV formation by ESCRT-II/ESCRT-III can be reconstituted *in vitro* with a GUV-based bulk-phase uptake assay. The activity of a minimal ESCRT-III/Vps4 system in artificial vesicle systems was first demonstrated by Wollert et al. (Wollert et al., 2009) where ILVs were generated in GUV parent vesicles and observed by microscopy. In addition, there are examples of more minimal sets of ESCRTs inducing ILV formation *in vitro*, such as Vps20/Snf7 only (Avalos-Padilla et al., 2018), and some minimal activity with moderate concentrations of Snf7 alone (Marklew et al., 2018), suggesting further scope for simplification of the system in a technological context.

**Figure 2:**
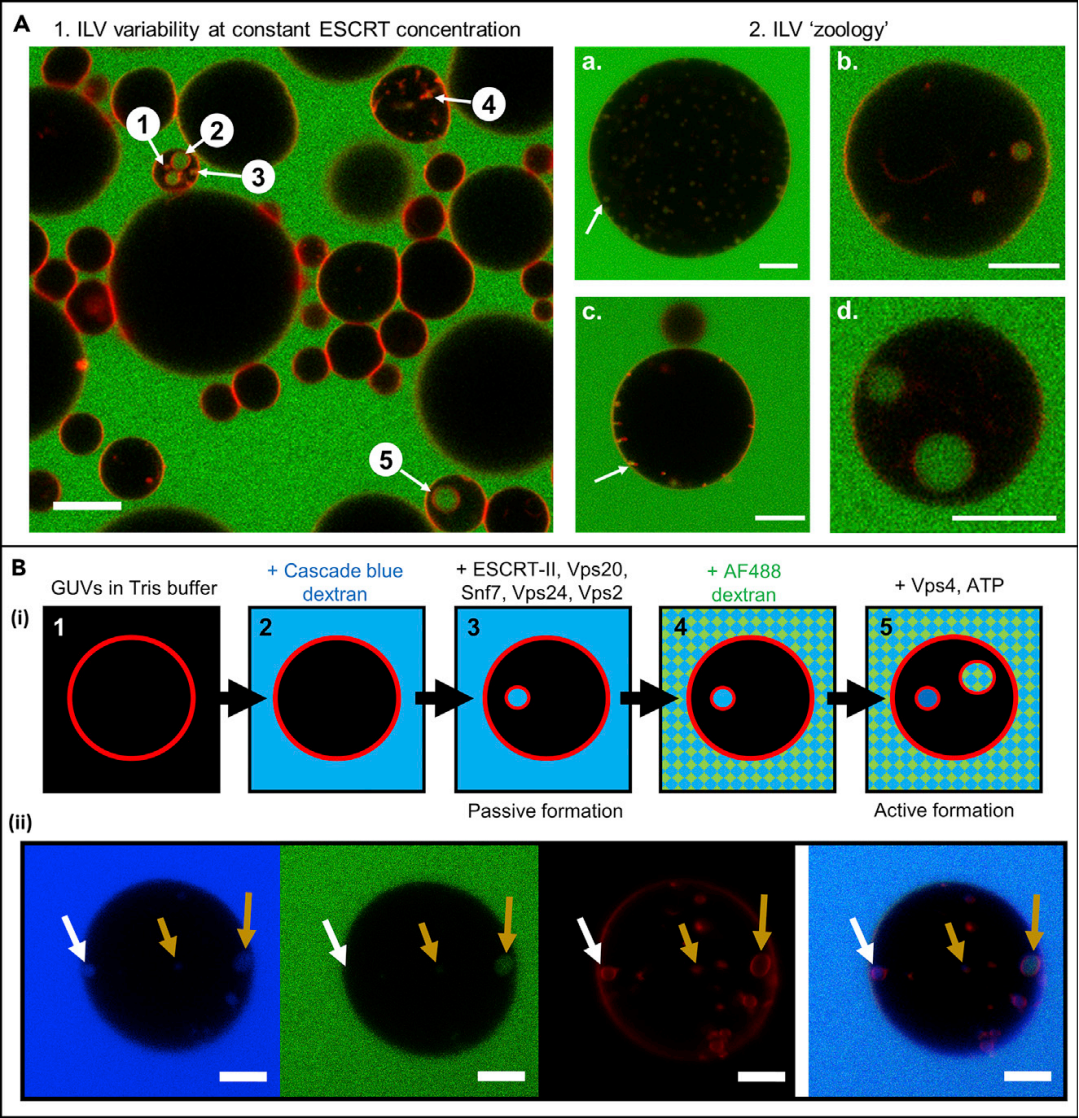
ESCRT-induced ILV Formation Is Characterised by a Bulk Phase Encapsulation Assay (A) A.1: Confocal microscope image of a cross section (3.1 μm) through a number of GUVs incubated with ESCRT-II, Vps20, Vps24, Vps2 (10 nM), and Snf7 (50 nM). A number of different ILV sizes are observed in the field of view (1–5). Red, Rhodamine-PE; green, Alexa Fluor 488-labeled dextran. Scale bar, 10 μm. A.2: ILV morphology, a range of commonly observed ILV states. (a) Many similarly sized ILVs plus buds, (b) few ILVs of varying dimensions, (c) “stalled” ILV buds with few or no coincident free-floating ILVs, (d) unusually large ILVs ∼5 μm in diameter. Red, Rhodamine-PE; green, Alexa Fluor 488-labeled dextran. All scale bars, 10 μm. (B) (i) Schematic of the ILV formation procedure. GUV membranes are labelled by a red Rhodamine lipid probes (1). Cascade Blue-labeled dextran was added to GUVs in Tris buffer (2), immediately followed by ESCRT-II, Vps20, Vps24, Vps2 (10 nM), and Snf7 (50 nM) (3). After a 20-min incubation period, Alexa Fluor 488-labeled dextran was added (4), immediately followed by Vps4 (10 nM) and ATP.MgCl_2_ (1 μM) (5). Thereby, ILVs containing only Cascade Blue dextran can be identified as having formed before the addition of Vps4 and ATP, and those that contain both dyes must have formed after addition. (ii) Confocal image of a GUV containing ILVs formed by active (ATP driven) and passive (no ATP) processes. Cascade Blue-labeled dextran (blue channel) was added immediately before incubation with ESCRT-II, Vps20, Vps24, Vps2 (10 nM), and Snf7 (50 nM). Alexa Fluor 488-labeled dextran (green channel) was added just before the addition of Vps4 (10 nM) and ATP.MgCl_2_ (1 μM) followed by a further period of incubation. White arrows indicate passively formed ILVs, and yellow arrows indicate actively formed ILVs. Membranes labeled with lissamine-Rhodamine-PE (red channel). 40× objective, scale bar, 10 μm.

The reconstitution of ESCRT activity *in vitro* opens the possibility of achieving finer control of ILV formation in artificial systems. Therefore we initially set out to investigate the relative stoichiometry of ESCRT-III components to underpin their influence on the size and number of ILVs that form within individual GUVs (Figure 1B). Interestingly, our initial experiments reveal wide variation in ILV size and number between different GUVs within an individual experiment (Figure 2A). Membrane mechanics are considered to be the likely source of these differences due to variability in tension across a single GUV population. Modulating membrane tension could therefore provide a further means to exert control over ILV formation and will also be investigated in this study.

Here we report on how changing the relative stoichiometry of the ESCRT-III subunits and the mechanical properties of the membrane regulate the size and number of ILVs formed in a GUV-based bulk-phase uptake assay (Figure 1B). These findings reveal a mechanism that regulates ESCRT activity *in vitro* and provide further insight into how they may function *in vivo*.

## Results

### “Passive” and “Active” Membrane Budding and Scission by ESCRT

ESCRT components were overexpressed and purified from appropriate plasmids (see Transparent Methods and Figure S1). We have quantified ESCRT activity using the bulk-phase uptake assay as described by Wollert and Hurley (Wollert et al., 2009), introducing a systematic variation in the ratio of key ESCRT-III in the reaction. In this assay, GUVs with an “endosome-like” lipid composition (Wollert et al., 2009) are incubated with the ESCRT-II complex (Vps22, Vps36, Vps25), and the core ESCRT-III subunits Vps20, Vps24, Vps2, and Snf7. Before the addition of proteins, a membrane-impermeable, fluorescent dextran (M_r_ ∼ 5 kDa, Cascade Blue labeled) is added to the GUV suspension to establish whether newly formed ILVs have encapsulated bulk phase (Figure 2A). Confocal microscopy (Figure 2B) is used to assess the number of ILVs, and the observed diameter of each ILV is recorded (observed diameter may not be the true maximum diameter of the ILV). Only ILVs visibly containing dextran fluorescence are counted, as this confirms their formation following addition of the proteins. The number of ILVs and the volume of the GUV lumens in the image is then used to calculate the number of ILVs per unit volume, the unit volume being taken as that of an average 20-μm-diameter GUV, hence “ILVs per GUV volume equivalent.” Controls containing only osmotically balanced buffer and fluorescent dextran give a minimal background level of ILVs formed within the experimental period (see “no protein control” in figure legends). The process of ILV formation observed in the absence of Vps4 and ATP is referred to as “passive – ESCRT only,” or simply “passive” because it identifies budding events that are independent of energy input from ATP hydrolysis, dependent only on ESCRT-III. In the context of *in vivo* ESCRT activity, this process would be considered to be off-pathway as recent evidence supports the requirement of the ATPase activity of Vps4 for efficient membrane deformation and scission (Adell et al., 2014, Adell et al., 2017, Mierzwa et al., 2017). A second fluorescent dextran (M_r_ ∼ 10 kDa, Alexa Fluor 488 labeled) can be added to this GUV population, followed by 20-min incubation with Vps4 and ATP.MgCl_2_. Newly formed ILVs in these conditions will contain blue and green fluorescence. The process of ILV formation driven by ATP hydrolysis by Vps4 is referred to as “active − + Vps4/ATP,” or simply “active.” Both passive and active formation of ILVs, induced by the action of ESCRTs, is observed in GUVs (Figure 2B (ii)).

### ILV Formation Efficiency Reaches a Maximum for a Specific Molar Ratio of Snf7

Snf7 oligomerization is central to ESCRT-III activity, with more Snf7 units present in the active complex than other ESCRTs (Wollert and Hurley, 2010). The relationship between ESCRT-III subunit stoichiometry and activity has been studied *in vivo* (Adell et al., 2017), whereas this is currently not well understood *in vitro*.

**Figure 3:**
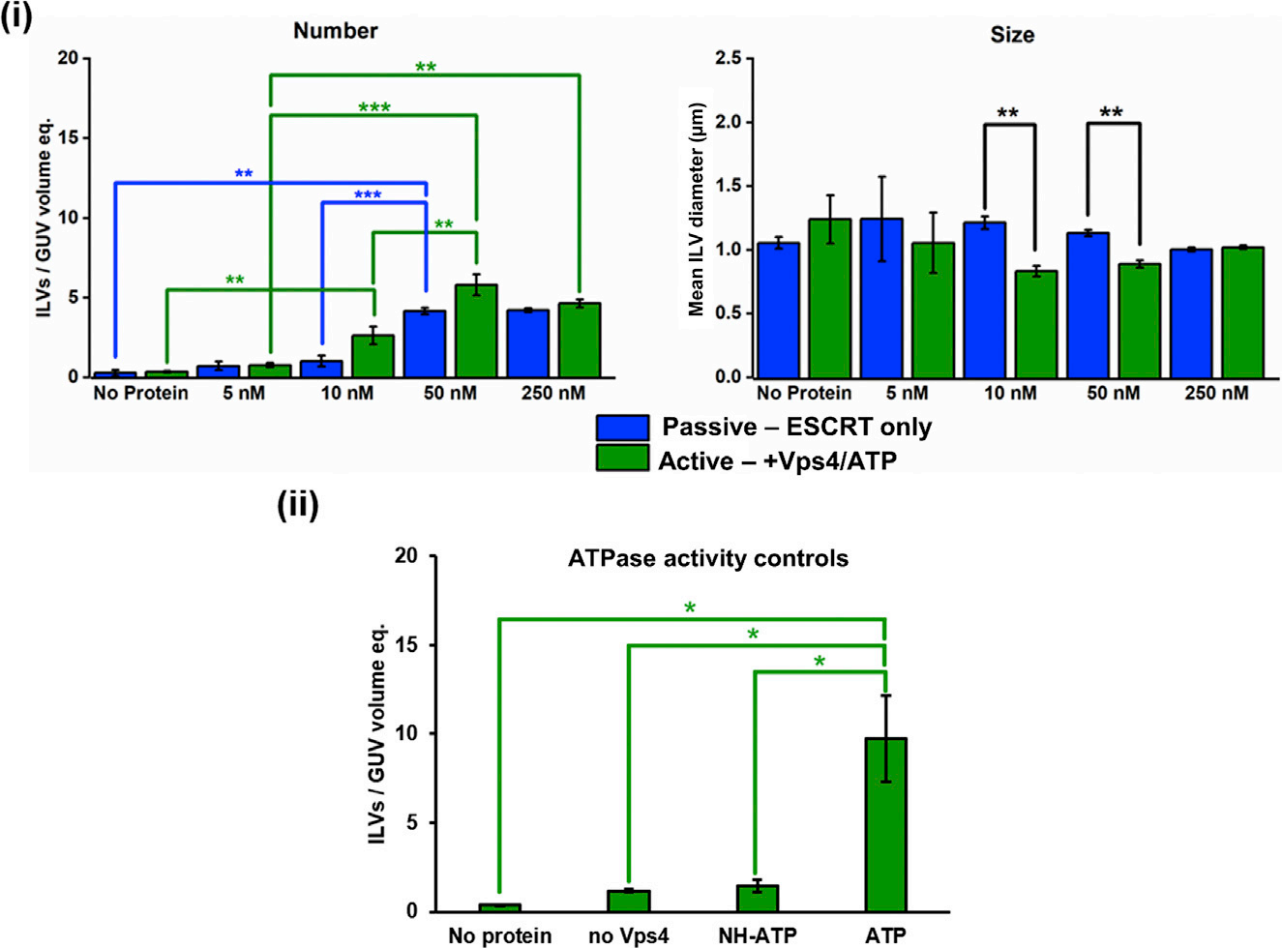
Snf7 Stoichiometry Bulk-Phase Encapsulation Assay; ILV Counting and Characterization by Confocal Microscopy ILVs containing fluorescent dye in their lumens after the addition of ESCRT proteins. (i) Number: number of ILVs per GUV volume equivalent; the number of ILVs observed per volume of a 20-μm-diameter sphere. Size: mean diameter of the observed ILVs. Blue: “passive” formation. ILVs containing Cascade Blue-dextran, after simultaneous addition of ESCRT-II, Vps20, Vps24, and Vps2 (10 nM) and Snf7 at the concentration stated on the x axis, but before the addition of 10 nM Vps4 and 1 μM ATP.MgCl_2_. Green, “active” formation. ILVs containing Alexa Fluor 488-dextran observed after the addition of 10 nM Vps4 and 1 μM ATP.MgCl_2_. Corresponding size histograms for each data point are presented in Figure S4. (ii) ATPase activity controls: number of “actively” formed ILVs (containing both blue and green dyes) per GUV volume equivalent. “No protein,” GUVs with no addition of protein incubated with both dextran dyes for the same time as the protein-containing experiments; “no Vps4,” all proteins added (50 nM Snf7, 10 nM others) + 1 μM ATP.MgCl_2_ with Vps4 omitted; “NH-ATP,” all proteins added (50 nM Snf7, 10 nM others) + 1 μM MgCl_2_ and the non-hydrolyzable ATP (“NH-ATP”) analog adenylyl-imidodiphosphate; “ATP,” all proteins (50 nM Snf7, 10 nM others) + 1 μM ATP.MgCl_2_. Osmotically relaxed vesicles were used in all experiments. Data are represented as the mean value ± standard error of the mean. Each data point is averaged from n = 3 independent experiments, each containing 100+ independent GUVs. Significance testing: one-way ANOVA with Bonferroni test, one-tailed, *p < 0.05, **p < 0.01, ***p < 0.001. No ESCRT control background rate = 0.067 ILVs per unit volume (standard error: 0.016).

Bulk-phase encapsulation assays are performed at a range of Snf7 concentrations (5–250 nM) and maintain a fixed concentration (10 nM) of each of the remaining ESCRT-II and ESCRT-III components (Figure 3). For both passive and active processes, 50 nM Snf7 provides the greatest activity in terms of number of ILVs observed, with no improvement at 250 nM. Interestingly, this is comparable to Snf7 being present at approximately up to five times the concentration of other ESCRT-III components in yeast (Adell et al., 2017, Wollert and Hurley, 2010).

A similar number of ILVs form during the passive and active processes, which may indicate “recycling” of the membrane-bound ESCRT components once and their reassembly into a similar number of active complexes. However, other explanations are plausible, and this may be complicated by changes and variability in GUV membrane tension in the second “active” round of ILV formation. ILV size is also found to be generally consistent across the full concentration range, with the only significant differences being found between active and passive processes at the 10 nM and 50 nM Snf7 concentrations, where ATP-fueled “active” formation favors slightly smaller ILVs, on average. Below 10 nM and above 50 nM Snf7 may be too little or too much protein, respectively, with respect to other ESCRT-III components to produce fully competent complexes.

The role of Vps4 ATPase activity in the active process is confirmed by the control experiments in Figure 3 (ii). Omission of Vps4 or replacement of ATP with a non-hydrolyzable ATP analog (adenylyl-imidodiphosphate) significantly supresses the active formation process. Nonetheless, a few Alexa Fluor 488-labeled dextran-containing ILVs are observed in each case. This low background rate of encapsulation may be the result of mechanical disturbance of the sample solution during the addition of the second round of proteins/ATP. Vps4 itself may possess some innate activity in the absence of ATP, which may account for the slightly greater number of “green” ILVs found in that case, but the difference between the “NH-ATP” and “no Vps4” controls is not found to be significant. Figure 3 (ii). “ATP” contains the full set of proteins plus ATP.MgCl_2_, at the same concentrations as Figure 3 (i) “50 nM,” but a higher number of ILVs per GUV volume equivalent is observed in these experiments. This variability is ascribed to variation in protein quality between expressed batches: all data presented on a single plot are obtained with the same batch of protein. This means that there are small differences between some of the absolute values and associated errors of ILVs formed under nominally identical conditions within the different figures because these were independent experiments performed with different protein batches.

### Membrane Tension Is the Dominant Factor in ILV Formation Efficiency and Size: Protein Stoichiometry Effects Are Minimal

Vps20 bridges ESCRT-III to ESCRT-II and is involved in the nucleation of Snf7 oligomers onto endosomal membranes *in vivo* (Fyfe et al., 2011, Henne et al., 2012). Vps20 possesses an N-terminal myristoylation that facilitates binding to membranes in general (Im et al., 2009); the ESCRT-II complex activates Vps20 by a mechanism including binding to the subunit Vps25 and generating initial invagination of the membrane (Boura et al., 2012). Activation of Vps20 leads to the recruitment and assembly of Snf7 and other downstream ESCRT-III proteins (Fyfe et al., 2011, Im et al., 2009). Nucleation is a critical step in the formation of ILVs, therefore Vps20 stoichiometry was examined for its influence on ILV size and number. Varying the Vps20 concentration relative to that of other ESCRT-III components should provide different numbers of potential nucleation sites for ESCRT-III assembly. A higher number of nucleation sites should result in a similarly higher number of ILVs, provided that there are sufficient other ESCRT-III components to form functional assemblies at each of these sites. The “dilution” of the available ESCRT-III components across a larger number of nucleation sites may also result in smaller Snf7 assemblies, which may give rise to smaller ILVs, if there is a relationship between the size of the complex and the resulting ILV. Furthermore, these experiments are performed with both high- and low-membrane-tension GUVs, where low-tension GUVs are subjected to an osmotic relaxation protocol, to assess the influence of membrane mechanics.

**Figure 4:**
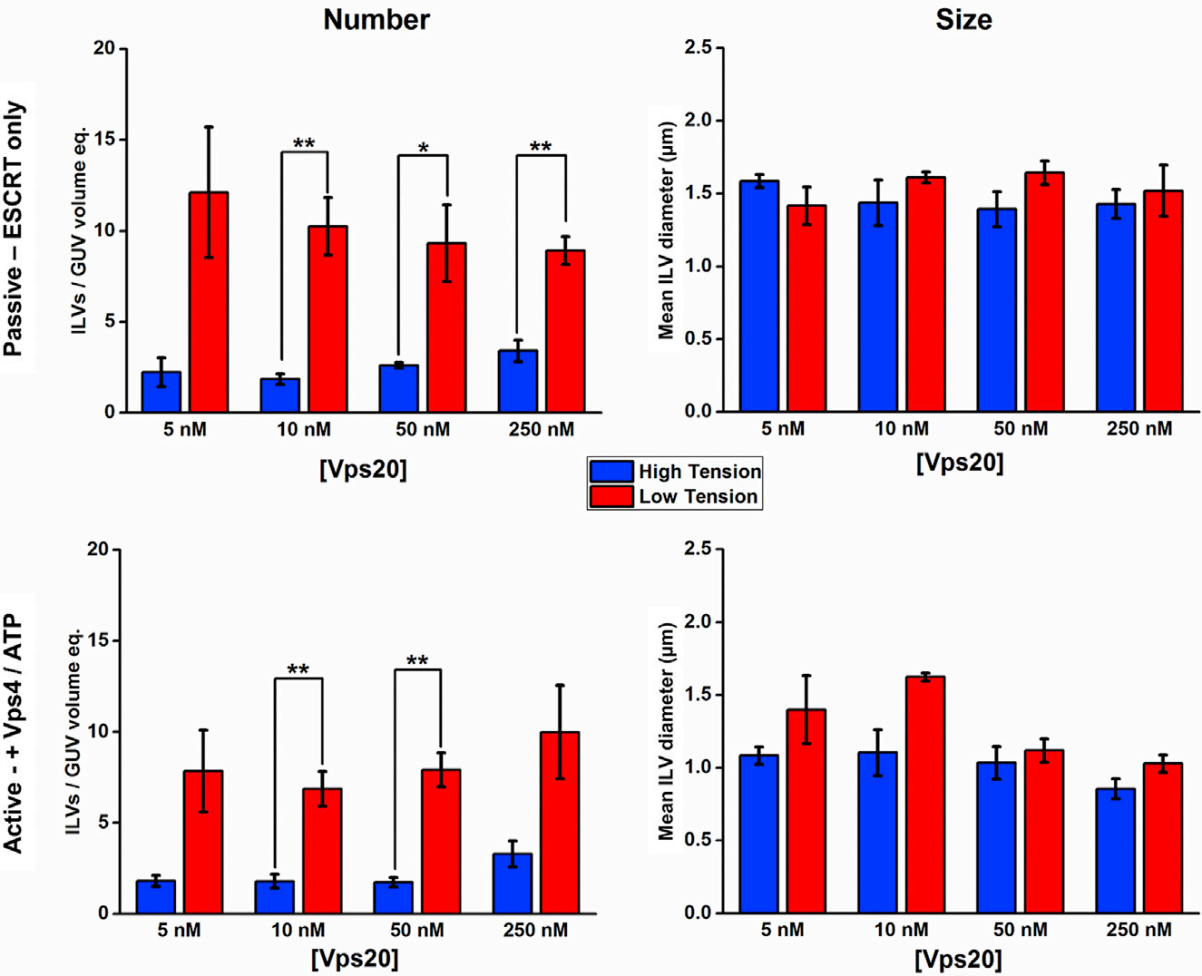
Vps20 Stoichiometry Bulk-Phase Encapsulation Assay; ILV Counting and Characterization by Confocal Microscopy ILVs containing fluorescent dye in their lumens after the addition of ESCRT proteins. Number: number of ILVs per GUV volume equivalent. Size: mean diameter of the observed ILVs. Blue, high-membrane-tension GUVs; red, low-membrane-tension GUVs. Passive: ILVs observed after simultaneous addition of ESCRT-II, Vps24, and Vps2 (10 nM), Snf7 (50 nM), and Vps20 at the concentration stated on x axis, but before the addition of 10 nM Vps4 and 1 μM ATP.MgCl_2_. Active: after the addition of 10 nM Vps4 and 1 μM ATP.MgCl_2_. Data are represented as the mean value ± standard error of the mean. Each data point is averaged from n = 3 independent experiments, each containing 100+ independent GUVs. Significance testing: one-way ANOVA with Bonferroni test, one-tailed, *p < 0.05, **p < 0.01. No ESCRT control background rate for low tension GUVs = 0.067 ILVs per unit volume (standard error: 0.016), for high tension GUVs = 0.054 ILVs per unit volume (standard error: 0.017). Corresponding size histograms for each data point are presented in Figure S5.

Across a 50-fold variation in concentration, the relative stoichiometry of Vps20 to other ESCRT subunits does not appear to have a significant effect on the number of ILVs observed (Figure 4 “number”), whereas a reduction in membrane tension results in 3–4 times the number of ILVs formed per unit volume for both active and passive activities. Neither Vps20 stoichiometry nor membrane tension have a significant effect on ILV size (Figure 4 “size”).

In a recent model of ESCRT-III assembly (Chiaruttini et al., 2015, Mierzwa et al., 2017, Saksena et al., 2009), Vps24 and Vps2 bind to the growing end of the Snf7 filament providing a limiting signal to filament growth by terminating Snf7 spiral elongation. Vps2 recruits the ATPase Vps4 to the ESCRT-III assembly to recycle the proteins and allow for multiple rounds of ILV formation. However, competing models have also recently suggested the formation of mixed Snf7, Vps2, and Vps24 filaments where each protein has a different preferred curvature in the spiral assembly (Chiaruttini and Roux, 2017). In this experiment, we test how varying Vps24 stoichiometry influences the efficiency of ILV formation, which may occur either by premature termination of ESCRT-III filament growth or alternatively, by modulating the stored elastic energy in spiral ESCRT complexes.

**Figure 5:**
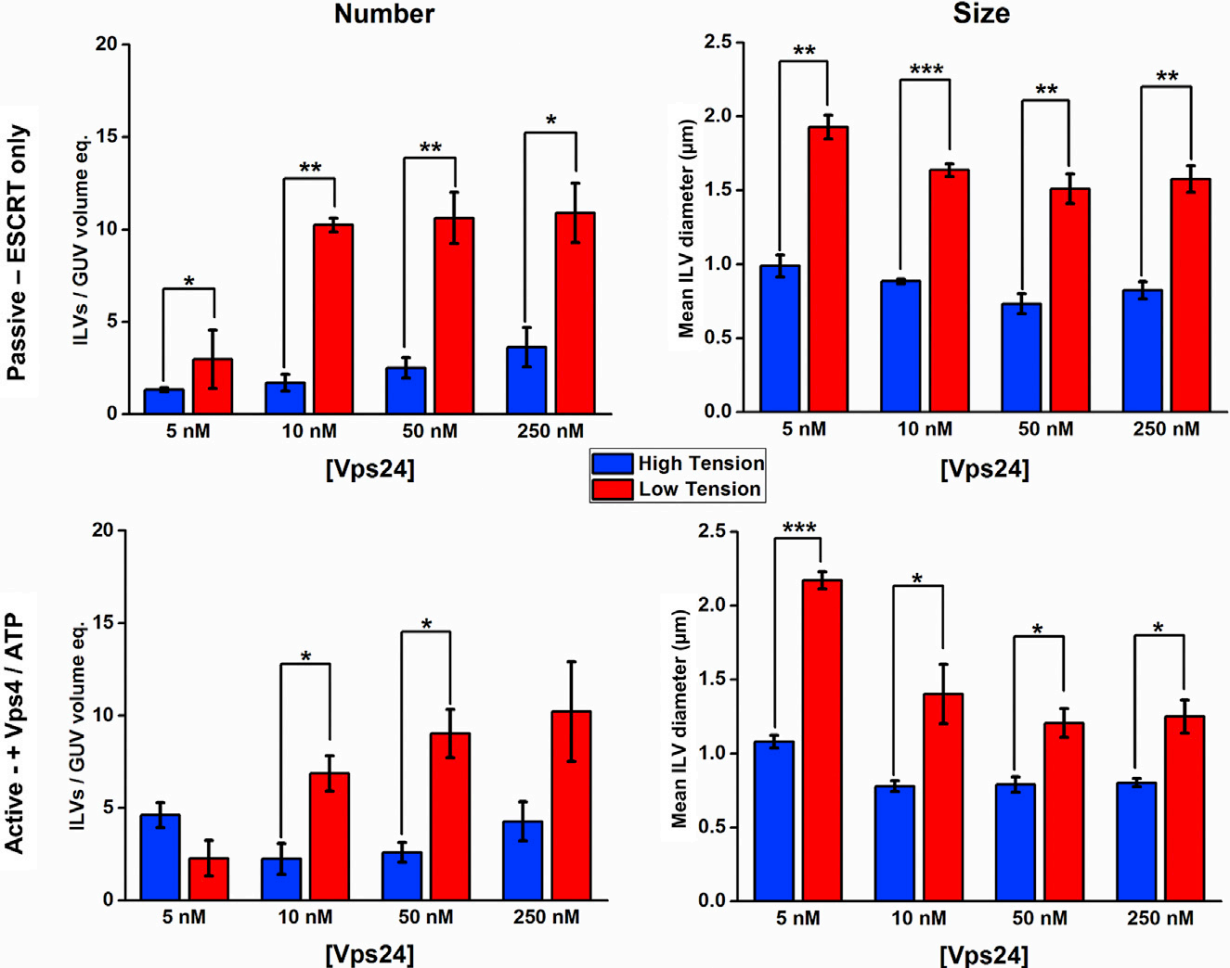
Vps24 Stoichiometry Bulk-Phase Encapsulation Assay; ILV Counting and Characterization by Confocal Microscopy ILVs containing fluorescent dye in their lumens after the addition of ESCRT proteins. Number: number of ILVs per GUV volume equivalent. Size: mean diameter of the observed ILVs. Blue, high-membrane-tension GUVs, red, low-membrane-tension GUVs. Passive: ILVs observed after simultaneous addition of ESCRT-II, Vps20, and Vps2 (10 nM), Snf7 (50 nM), and Vps24 at the concentration stated on x axis, but before the addition of 10 nM Vps4 and 1 μM ATP.MgCl_2_. Active: after the addition of 10 nM Vps4 and 1 μM ATP.MgCl_2_. Data are represented as the mean value ± standard error of the mean. Each data point is averaged from n = 3 independent experiments, each containing 100+ independent GUVs. Significance testing: one-way ANOVA with Bonferroni test, one-tailed, *p < 0.05, **p < 0.01, ***p < 0.001. No ESCRT control background rate for low-tension GUVs = 0.067 ILVs per unit volume (standard error: 0.016), for high-tension GUVs = 0.054 ILVs per unit volume (standard error: 0.017). Corresponding size histograms for each data point are presented in Figure S6.

Overall, we see a weak dependence of Vps24 stoichiometry on the efficiency of ILV formation in a similar fashion to what was observed for Vps20 (Figure 5), where the effects of membrane tension dominate the actions induced by stoichiometric changes of Vps24 with a 3- to 5-fold increase in ILV number in the low-tension regime. However, at the lowest Vps24 concentration (5 nM), there appears to be an inhibitory effect on ILV number in the low-tension GUV population, but in high-tension samples, no Vps24 concentration effect on number is observed. GUVs in the low-tension regime also display an effect of Vps24 stoichiometry on the size of ILV produced, with the lowest Vps24 concentration producing significantly larger mean ILV diameter for both active and passive formation, suggestive that increased capping of the complex by Vps24 favors smaller ILV sizes. Furthermore, we see a significant increase in ILV size in the low-tension regime in these experiments, indicating that lower mechanical resistance to membrane deformation facilitates more membrane, on average, being removed from the parent GUV in an individual ILV formation event.

Taken together, our data show that membrane tension plays a major role in regulating ESCRT-driven ILV formation.

### Membrane Tension Negatively Regulates ILV Formation by ESCRT-III

The deformation of a membrane during ESCRT-induced ILV budding would encounter greater mechanical resistance on higher tension membranes. The energetic input to achieve this may be derived from the assembly of ESCRT-III components, primarily Snf7 filaments, which are directly implicated in driving membrane budding. Although the role of Vps4, a mechanoenzyme, has been suggested to be in the scission of ILV buds from their parent membrane through the disassembly of stabilizing Snf7 filaments, some published results have suggested that ESCRT complexes only exert measurable forces on membranes during ATP-driven Vps4 activity (Schöneberg et al., 2018).

Our data reveal a strong, significant dependence of the efficiency of ILV formation on membrane tension, implying that typical membrane tensions in GUVs are sufficient to compete with the energetic driving force of membrane deformation exerted by the ESCRT complex. Therefore regardless of the mechanism or magnitude of the force exerted, there must exist a maximum membrane tension that is able to undergo ESCRT-induced ILV budding. A relationship between ILV size and membrane tension is also plausible as the magnitude of membrane deformation that can be achieved during budding may be modulated by tension. Any such effects resulting from resistance to membrane deformation will necessarily be concurrent with any effects resulting from transient membrane curvature. As assemblies of ESCRT-II and Vps20 are known to have an increased affinity for regions of membrane curvature (Fyfe et al., 2011), the transient curvature changes from membrane undulations that are more pronounced in low-tension membranes may also enhance the affinity of ESCRT-II/Vps20 complexes for the membrane.

Interestingly, we find that the suppression of ILV formation is of a similar extent in both the passive and active processes. This implies that the inhibitory mechanism is not significantly dependent on Vps4 and ATP. Therefore we hypothesize that either the complex exerts a similar magnitude of deformation force on the membrane in both the active and passive processes or the increased recruitment of ESCRTs to the membrane due to enhanced undulatory curvature at low membrane tension is the dominant factor. As a recent report shows measurable membrane deformation forces only occur due to Vps4 activity (Schöneberg et al., 2018), this lends favor to the latter interpretation. Interestingly, simulations have previously reported analogous tension-dependent assembly of N-BAR domains on membranes (Simunovic and Voth, 2015).

Flicker spectroscopy experiments are used to quantify the distribution of membrane tensions (σ) in GUV populations in our low- and high-tension regimes, before and after incubation with ESCRT proteins (Figures 4, S2, and S3.).

GUVs are found to have high membrane tension following initial electroformation. Addition of the ESCRT complex resulted in a narrowing of the distribution of membrane tensions toward the high-tension end of the initial distribution (Figure S2). This implies that the lower-tension vesicles within the initial population have increased their tension due to their interaction with ESCRTs. This is likely due to ILVs forming within the less tense vesicles of the high-tension population and a significant number of GUVs where no ILVs have formed are observed in these samples, suggesting that their tension might be too high for the ESCRT complex to act upon them. Some GUVs in these samples are too tense to be quantified by the full flicker analysis as the data for higher *q* modes became noisy owing to their small amplitude. Therefore the mean square amplitudes of the longest wavelength undulatory mode (n = 3) are analyzed as a proxy for membrane tension where smaller mean square amplitudes correspond to a higher membrane tension (Amjad et al., 2017).

**Figure 6:**
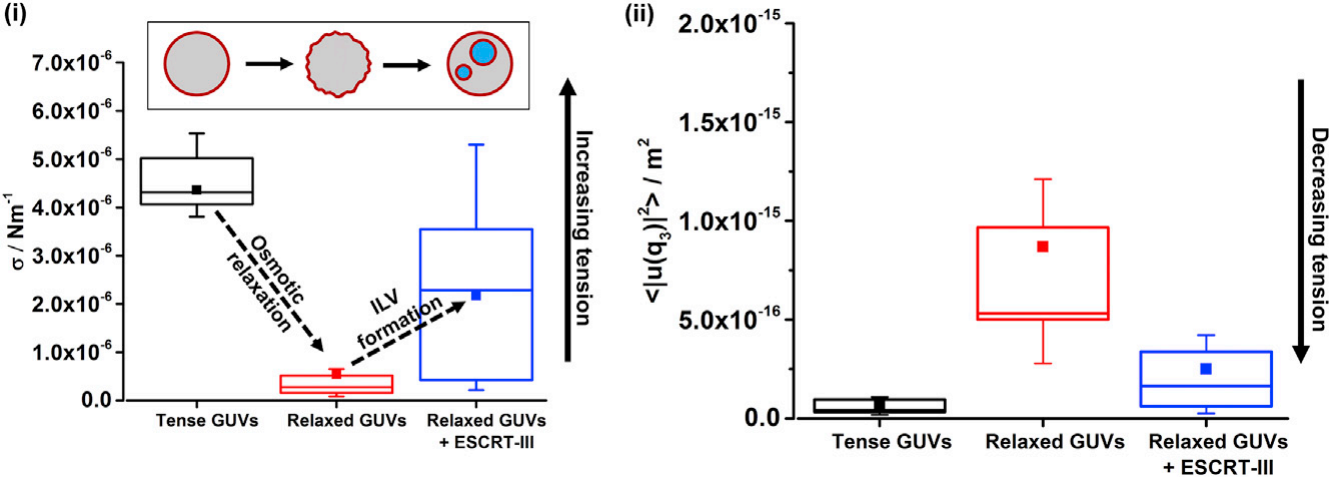
ESCRT Activity Increases Membrane Tension (i) The distribution of membrane tension σ in GUV samples. (ii) Distributions for the values of the amplitude of the third vibrational mode for the same GUVs. “Tense” GUVs were those that were not exposed to an osmotic gradient. The membrane tension of osmotically “relaxed” GUVs was measured before (pre-protein) and after (post-protein) the addition of the ESCRT proteins to assess the effect of ILV formation on membrane tension. Box descriptions: ■, mean values; line termini correspond to the minimum and maximum observed values; the upper and lower bounds of the boxes correspond to the 75^th^ and 25^th^ percentile values, respectively; and the inner line corresponds to the 50^th^ percentile. n = 10. See also Figures S2 and S3.

The mechanical coupling between membrane tension and ESCRT function is most evident in the low-tension regime. As expected, the osmotic relaxation procedure reduces the mean membrane tension by about an order of magnitude and also narrows the range of tension values across the population (Figure 6 “relaxed GUVs”). As discussed above, the error of the full analysis of flicker spectrum increases at higher tension, therefore we present both the absolute tension measurement and the mean-square amplitude of the third mode in Figure 6 to demonstrate that both analysis methods demonstrate the same data trends. Similarly, due to increasing tension after protein addition, bending modulus (*κ*) values could only be reliably obtained for osmotically relaxed GUVs before the addition of the ESCRT proteins. Relaxed GUVs were found on average to have *κ* = 24.5 *k*_*B*_*T*, which agrees with those typically reported for 1-palmitoyl-2-oleoyl-glycero-3-phosphocholine (POPC) and cholesterol membranes (Dimova, 2014, Henriksen et al., 2006).

ESCRT activity increases membrane tension. Further flicker spectra are obtained after incubation with ESCRT proteins, using our standard procedure (50 nM Snf7; 10 nM ESCRT-II, Vps20, Vps24, Vps2, and Vps4; 1 μM ATP.MgCl2, 20-min incubation) (Figure 6. “relaxed GUVs + ESCRT-III”). The mean tension after ILV formation increases to an intermediate value between the “tense” GUVs and those that have been osmotically relaxed “pre-protein.” This increase in membrane tension will act to make further ILV formation more difficult, providing a mechanism of negative feedback to self-regulate ESCRT activity. Notably, the “post-protein” tension range does not reach the highest tension values observed in the initial electroformed GUV population, suggesting that in these cases, ILV formation is not limited by the membrane mechanics and further ILV formation is likely possible upon addition of more ESCRT-III or further active recycling of the existing components.

## Discussion

Here we have shown that ESCRT-III activity is regulated by the tension of the membrane substrate, using an *in vitro* model membrane system. Our work recapitulates ESCRT-mediated membrane budding and scission in the GUV system and shows that membrane tension significantly dominates over any attempt to control ILV formation by varying the ratio of the ESCRT-III assembly reaction. Increased membrane tension through removal of excess membrane surface area provides a negative feedback mechanism that eventually suppresses membrane remodeling by ESCRT-III.

Based on a model wherein individual ESCRT constituents promote nucleation (Vps20), growth (Snf7), and termination (Vps24) of the ESCRT filaments, we hypothesized that variation of relative ratio between subunits may affect the efficiency of ILV formation and ILV size. Nonetheless, we observed minimal effects on ILV formation efficiency and size when varying Vps20 and Vps24 concentration across a 50-fold concentration range. Therefore, ILV formation under these conditions is weakly dependent on Vps20 and Vps24. This suggests that either membrane mechanics strongly dominates the effects of nucleation and termination in our experimental regime or there is a narrow range of stoichiometric composition where functional ESCRT complexes form, giving rise to a weak dependence on the concentration of a single component. We consider the latter scenario to be most likely given that ILV formation in relaxed vesicles is not limited by membrane mechanics at the typical protein concentrations used (Figure 6).

Mechanistic models of ESCRT activity revolve around the formation of circular or spiral structures on membranes that drive membrane invagination (Chiaruttini et al., 2015), constricting and stabilizing ILV bud necks (Adell et al., 2014) before scission by disassembly of the oligomer (Hurley and Hanson, 2010). Nonetheless, membrane curvature drives the recruitment of specific ESCRT subunits (Castro-Gomes et al., 2014) and membrane undulations, amplified at low tension, have been implicated in promoting self-assembly of other curvature-generating proteins at the membrane (Simunovic and Voth, 2015). Hence a more complete model for ESCRT-mediated remodeling activity includes the membrane as an active participant. Here, we have elucidated a regulatory role for membrane mechanics in preventing runaway overactivity of the ESCRT complex via a negative feedback mechanism.

Minimal model systems provide clues to the biological mechanisms and interactions of the reconstituted components, allowing hypotheses to be generated about the more complex biological scenario. ESCRTs perform a wide variety of membrane remodeling functions within the cell (Hurley, 2015) with some of these responding directly to changes in membrane tension, such as membrane abscission during cytokinesis, whereby relaxation of membrane tension promotes recruitment of ESCRT-III to the intercellular bridge (Lafaurie-Janvore et al., 2013). In the endocytic pathway, fusion of endosomes may provide excess membrane that lowers tension and could therefore facilitate generation of internal vesicles by ESCRT-III, suggestive of a homeostatic relationship between endosomal fusion and formation of vesicles within the MVB (Skjeldal et al., 2012). ESCRTs also play an important role in plasma and nuclear envelope membrane repair, where a loss in tension caused by membrane impairment could contribute to the rapid recruitment of the complex to the site of damage. Notably, the plasma membrane structures known as caveolae, and their associated proteins, act as a store of excess membrane area that provides a stress-responsive buffer of membrane tension, and their involvement in numerous signaling pathways demonstrates the sensitivity of cellular processes to changes in membrane tension (Parton and del Pozo, 2013).

Membrane tension could provide a general means of regulating ESCRT activity. Plasma membrane tension has been reported in the low tens of μN m^−1^ range (Sens and Plastino, 2015), which is above the range of values observed in our GUV membranes. This may suggest that ESCRT remodeling activity is generally inhibited until local events at the membrane lower tension and promote ESCRT reactions. Cellular membranes lacking cytoskeleton support, such as blebs, have tensions typically in the μN m^−1^ range (Sens and Plastino, 2015), matching the higher end of our measured values. Accordingly, our data show a significant increase in efficiency of ESCRT activity (as measured by efficiency of ILV formation) associated with a change in mean tension from 4.8 μN m^−1^ to 0.6 μN m^−1^. These represent physiologically relevant values of membrane tensions supporting the inference of our model for ESCRT activity regulation by membrane mechanics not only *in vitro* but also *in vivo*.

Our primary interest is to develop the ESCRT proteins as a means of directing compartmentalization in artificial cell systems, ultimately to enable dynamic control over their compartmentalized structures. Although the fundamental ILV-forming activity of ESCRT-III in GUVs is well established, our intention is to determine means of exerting finer control over compartment parameters, such as size, number, and cargo encapsulation. Membrane tension provides a promising control parameter to regulate ILV formation in GUVs and is suggestive of strategies that would allow multiple rounds of ILV formation to provide numerous distinct artificial organelles within GUV artificial cells.

### Limitations of the Study

Our study clearly demonstrates that membrane tension acts to regulate ILV formation by ESCRT in giant vesicle artificial cells. This tension control is most evident in the number of ILV compartments that are formed. We found that there is little control over the size of the ILV compartments, hence alternative approaches are required if compartment size would be an important parameter to regulate. Stoichiometry of ESCRT components appears to have a weak effect on ILV formation efficiency in our experimental conditions; however, this does not completely rule out stoichiometric control as this may exist in a different concentration regime. The parameter space for varying the concentrations and relative stoichiometries of ESCRT components is extremely broad and unfeasible to fully cover in a single study. We propose that the regulation of ESCRT activity by membrane mechanics observed *in vitro* will have relevance to understanding of ESCRT function *in vivo*. Naturally, further studies designed to elucidate this phenomenon in the complex cellular environment are required to confirm these predictions.

## Methods

All methods can be found in the accompanying Transparent Methods supplemental file.

## Data and Software Availability

The data files supporting this manuscript are available online in the Research Data Leeds repository at https://doi.org/10.5518/549.

## Supplemental Information

Document S1. Transparent Methods and Figures S1–S6
