# Supplementary material for: In Vitro Membrane Remodeling by ESCRT is Regulated by Negative Feedback from Membrane Tension
Source: iScience. 2019 Apr 20;15:173–84. doi: 10.1016/j.isci.2019.04.021 (PMC6503128; doi:10.1016/j.isci.2019.04.021)
Supplement: Document S1. Transparent Methods and Figures S1–S6 [file mmc1.pdf]

ISCI, Volume *15*

Supplemental Information

In Vitro Membrane Remodeling by ESCRT is Regulated by Negative Feedback from Membrane Tension

Andrew Booth, Christopher J. Marklew, Barbara Ciani, and Paul A. Beales

A

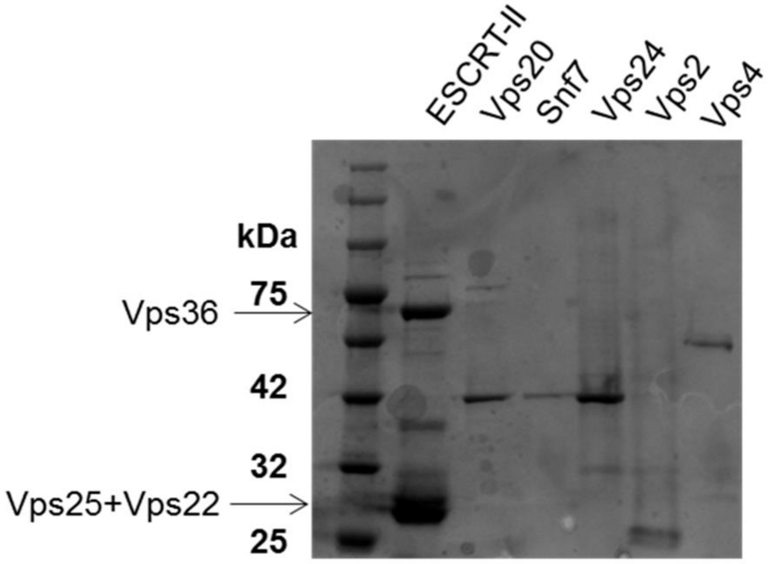

B

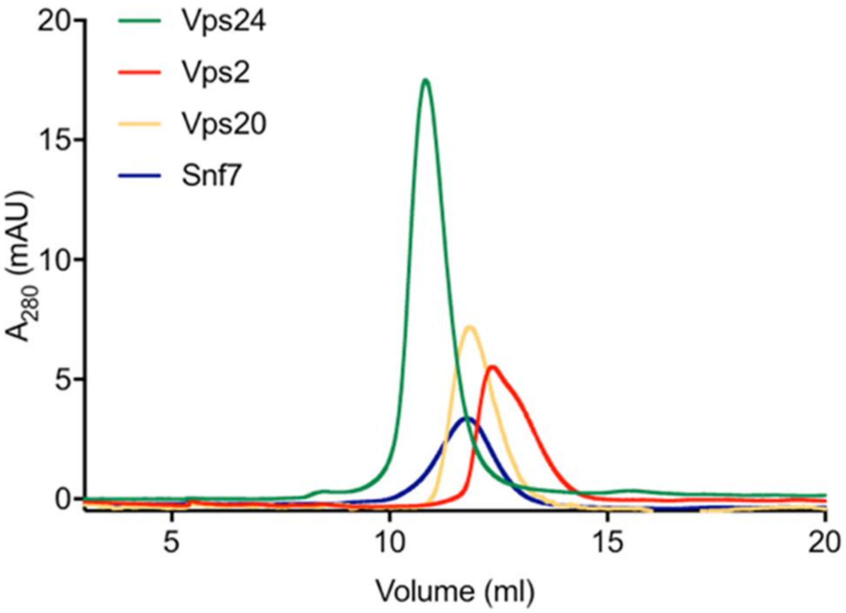

Supplementary Figure S1. Related to Figure 1. **A:** comassie-stained 10% SDS-PAGE of purified ESCRT-II and ESCRT-III proteins. **B:** size-exclusion chromatograms of Vps20, Snf7, Vps24, and Vps2. Purified proteins were run on a Superdex 75 (10/300 GL) column (GE Healthcare) at 0.8 ml min^{-1}. The absence of large oligomeric species was judged by the presence of a single peak corresponding to a low molecular weight.

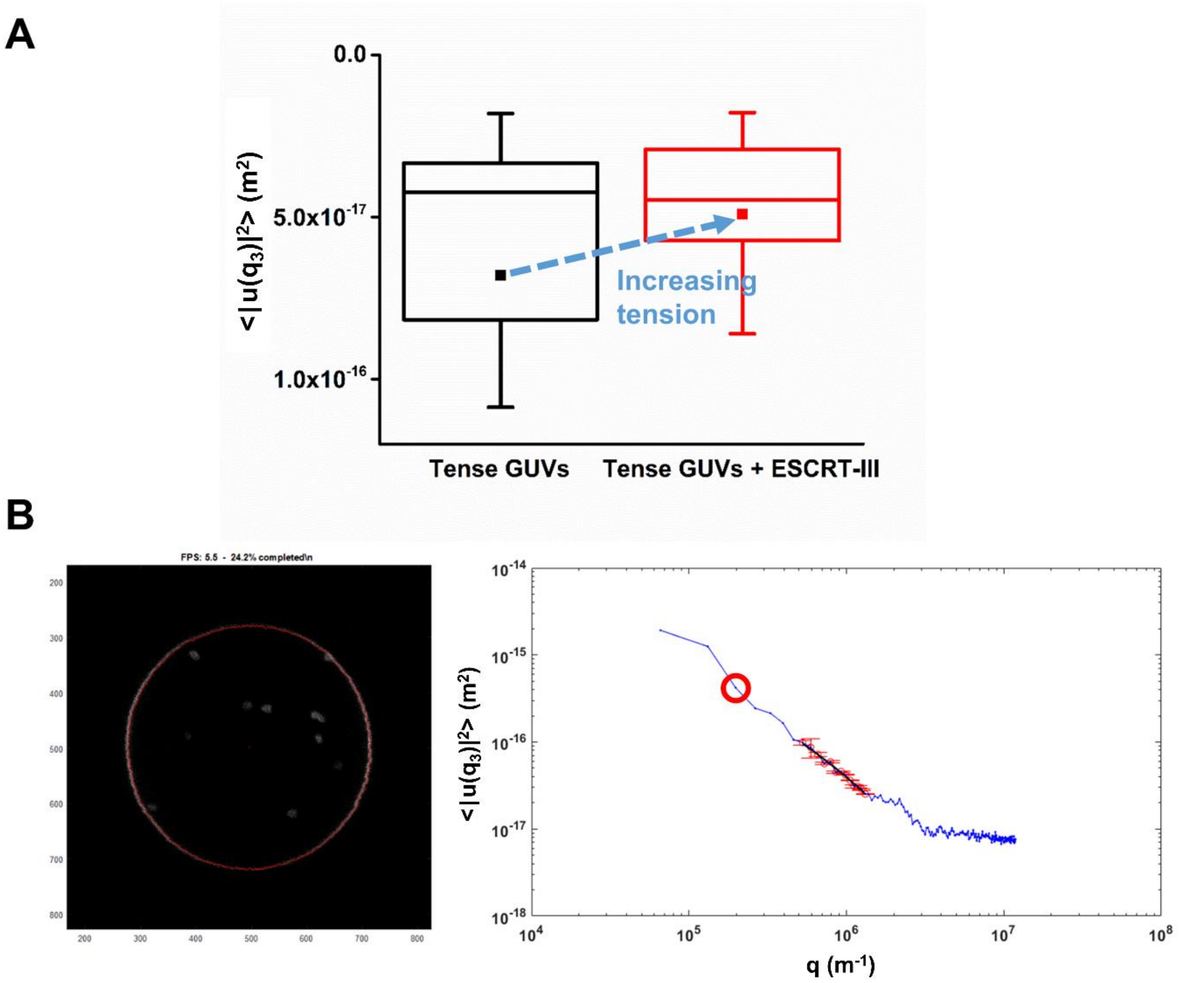

Supplementary Figure S2. Related to Figure 6. **A:** The distribution of values for the mean squared amplitude of the third undulatory mode $<|u(q_3)|^2>$ of tense GUVs (that have not been subjected to an osmotic gradient), before and after the addition of protein. Box descriptions: ■ – mean values, line termini correspond to the minimum and maximum observed values, the upper and lower bounds of the boxes correspond to the 75th and 25th percentile values respectively and the inner line, the 50th percentile. Lower values of $<|u(q_3)|^2>$ correspond higher to membrane tension values (σ). '**Tense GUVs'**: GUVs before addition of ESCRT proteins. **'Tense GUVs + ESCRT-III'**: GUVs after the addition of ESCRT-II, Vps20, Vps24, and Vps2 (10 nM), Snf7 (50 nM) and Vps4 (10 nM) and ATP.$MgCl_2$ (1 µM). Note that the y-axis is inverted so that higher tension data appear near the top of the graph. Box descriptions: ■ – mean values, line termini correspond to the minimum and maximum observed values, the upper and lower bounds of the boxes correspond to the 75th and 25th percentile values respectively and the inner line, the 50th percentile. n=10. **B:** An example flicker spectrum for membrane undulation tracking, resulting power spectrum and fitting to **Equation 1.** Where $<|u(q_x)|^2>$ is the mean squared amplitude of a given vibrational mode.The datapoint corresponding to the third vibrational mode, used to estimate membrane tension for very tense membranes, is highlighted with a red circle.

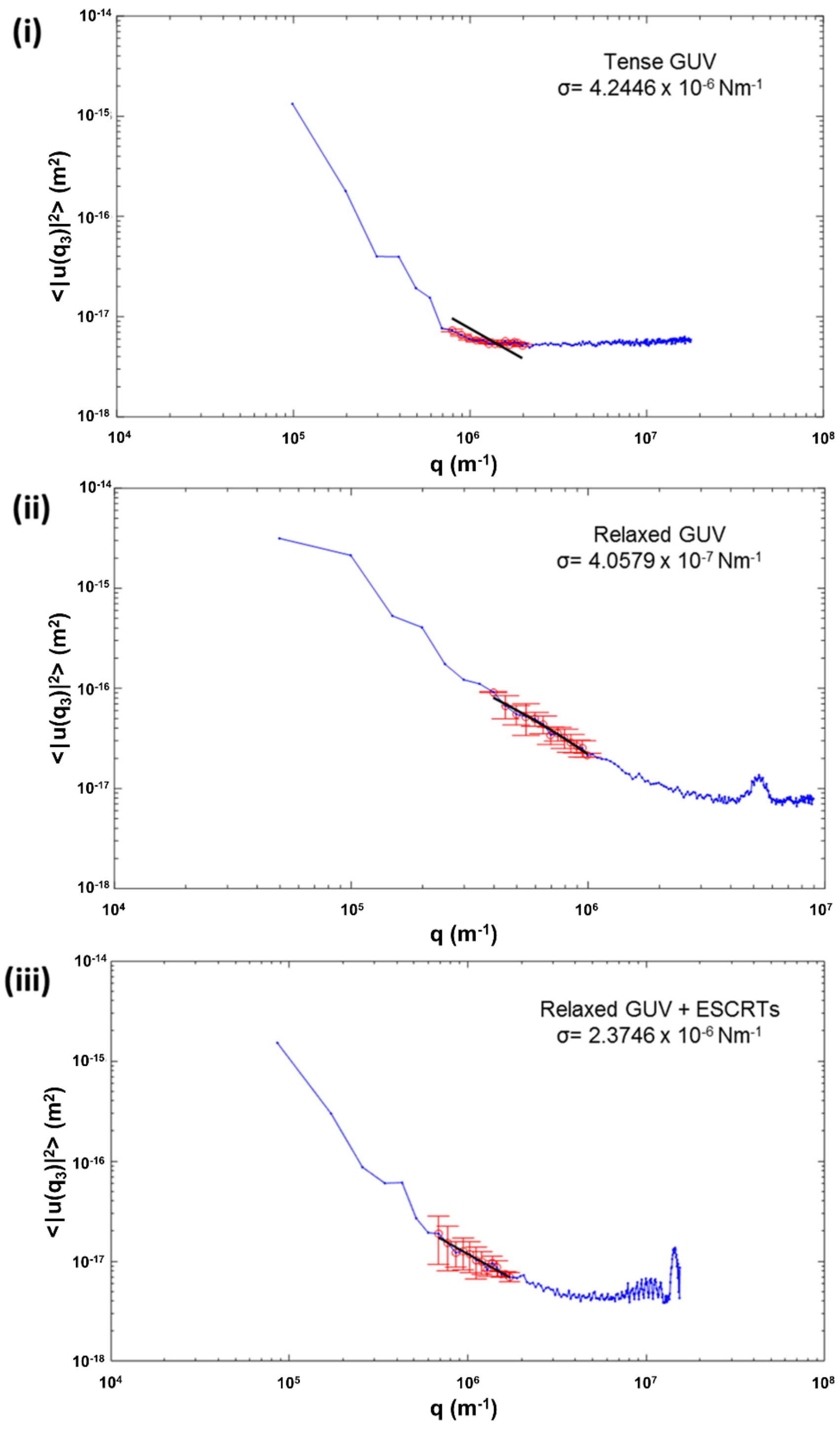

Supplementary Figure S3. Related to Figure 6. Example flicker spectra for key conditions in main paper **Figure 6**. **(i):** Tense GUVs, **(ii):** Osmotically relaxed GUVs, **(iii):** Osmotically relaxed GUV after ESCRT-induced ILV formation. Poor fitting to the model is observed for the exemplar highly tense GUV spectrum given in **S3(i),** necessitating examination of amplitude of the third undulatory mode (see **Figure 6(ii)** and **S2)**

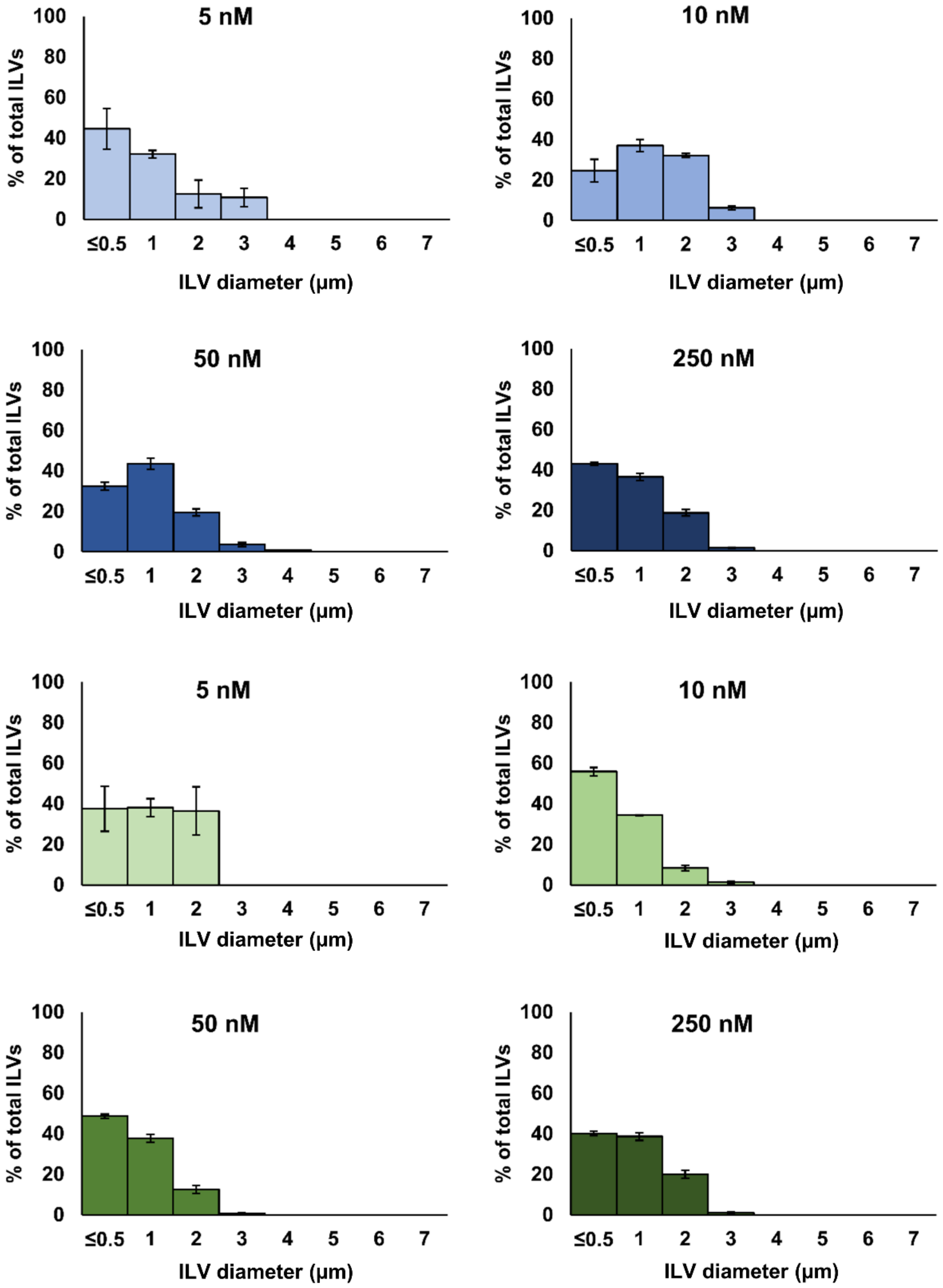

Supplementary Figure S4. Related to Figure 3. Histograms of observed ILV diameters as a percentage of the total number of ILVs at different Snf7 concentrations with 10 nM each of ESCRT-II, Vps20, Vps24, Vps2 (plus 10 nM Vps4 and 1 µM ATP.$MgCl_2$ for active formation) (see Figure 3). **Blue bars** = passive formation, **green bars**= active formation. Error bars are +/- the standard error of the mean.

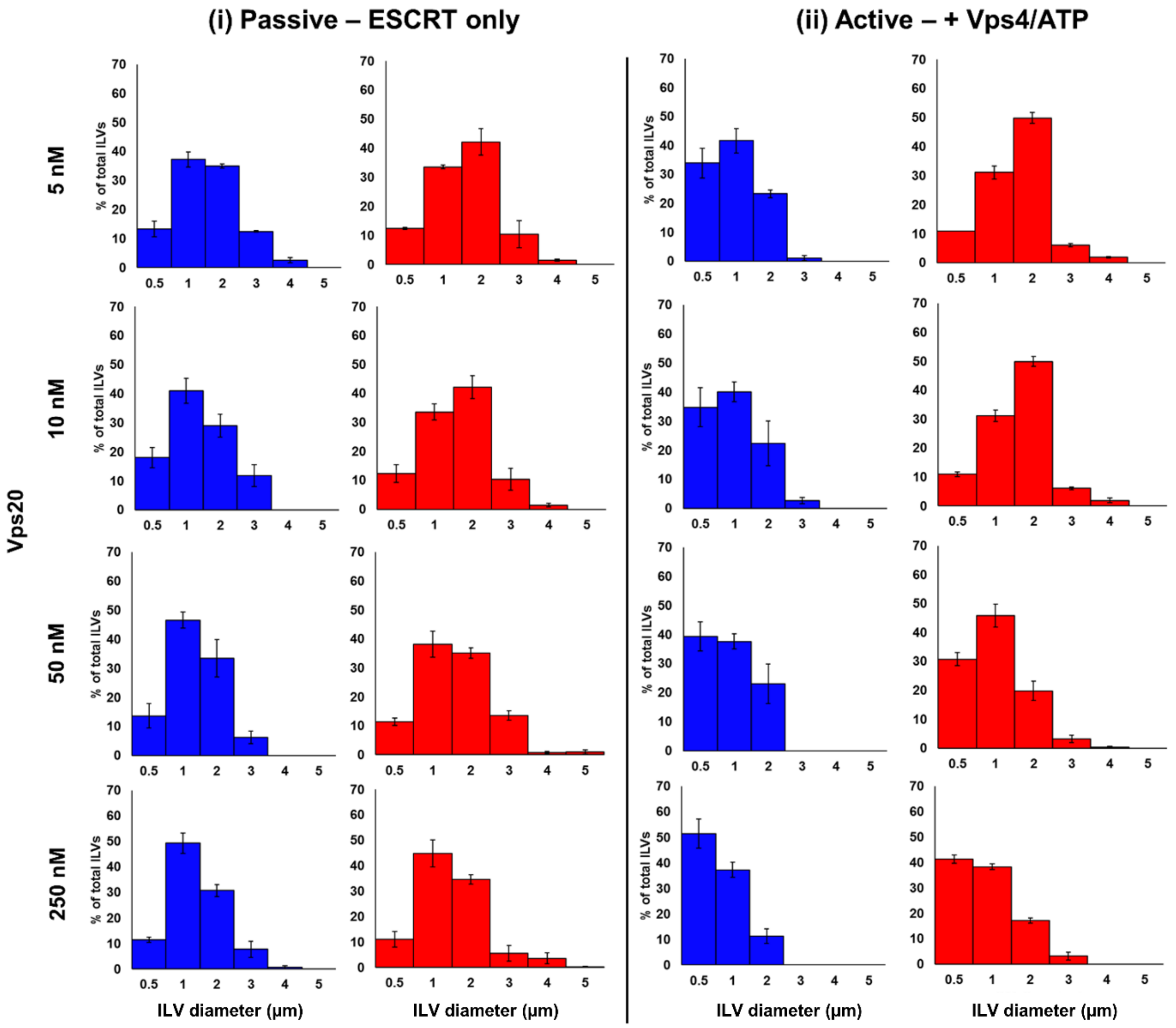

Supplementary Figure S5. Related to Figure 4. **(i)** Histograms of observed ILV diameters as a percentage of the total number of ILVs at different Vps20 concentrations for passive ILV formation conditions (see **Figure 4**): 10 nM each of ESCRT-II, Vps24, Vps2, Vps2. **Blue bars:** high membrane tension GUVs, **Red bars:** low membrane tension GUVs. **(ii):** Histograms of observed ILV diameters as a percentage of the total number of ILVs at different Vps20 concentrations under active conditions: 10 nM each of ESCRT-II, Vps24, Vps2, Vps2 + 10 nM Vps4 and 1 µM ATP.$MgCl_2$ (see **Figure 4**). **Blue bars:** high membrane tension GUVs, **Red bars:** low membrane tension GUVs. Error bars are +/- the standard error of the mean.

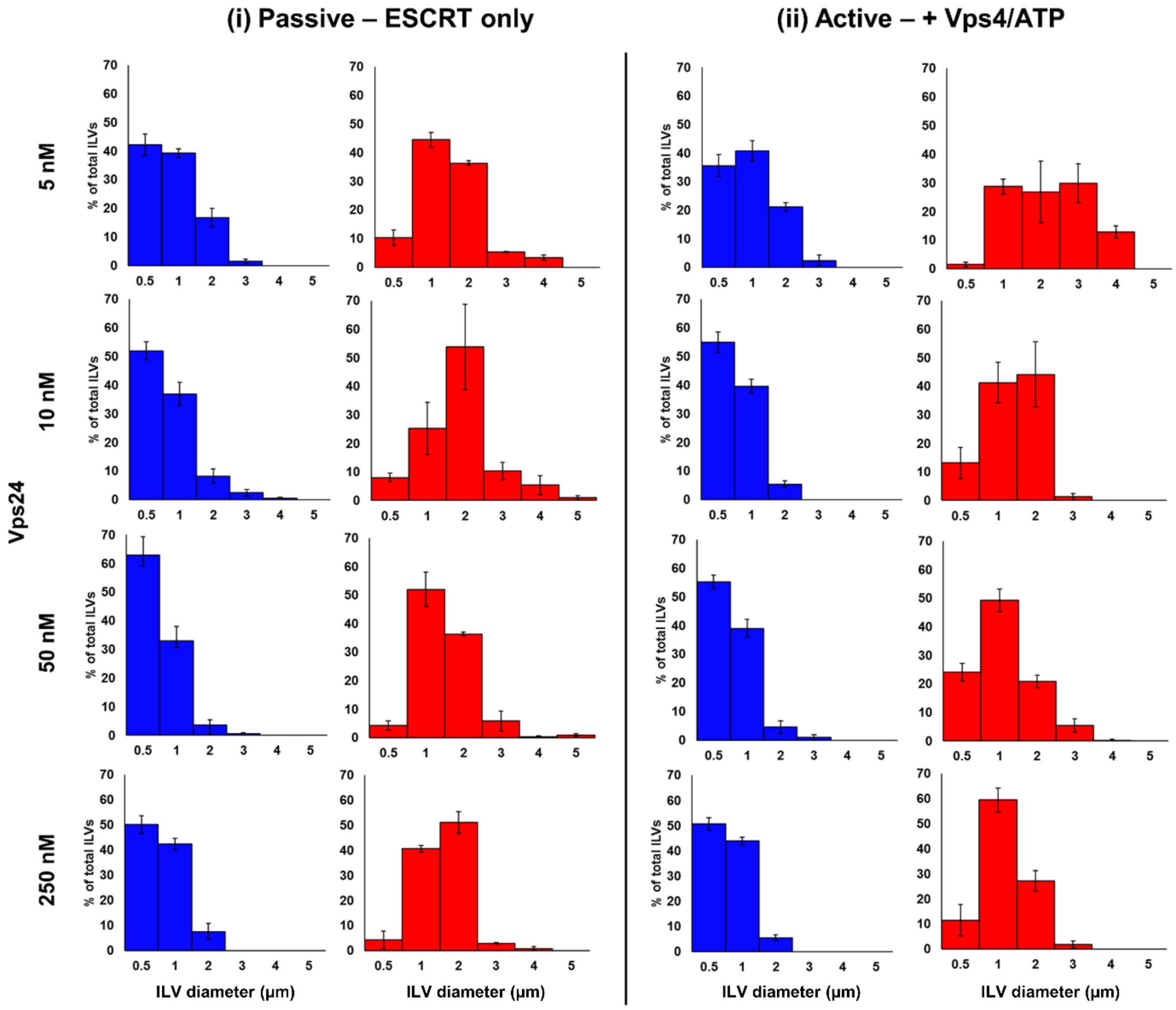

Supplementary Figure S6. Related to Figure 5. **(i)** Histograms of observed ILV diameters as a percentage of the total number of ILVs at different Vps24 concentrations for passive ILV formation conditions (see **Figure 5**): 10 nM each of ESCRT-II, Vps20, Vps2, Vps2. **Blue bars:** high membrane tension GUVs, **Red bars:** low membrane tension GUVs**. (ii):** Histograms of observed ILV diameters as a percentage of the total number of ILVs at different Vps24 concentrations under active conditions: 10 nM each of ESCRT-II, Vps20, Vps2, Vps2 + 10 nM Vps4 and 1 µM ATP.$MgCl_2$ (see **Figure 5**). **Blue bars:** high membrane tension GUVs, **Red bars:** low membrane tension GUVs. Error bars are +/- the standard error of the mean.

Transparent methods

Protein over-expression and purification

The plasmids for overexpression of Saccharomyces cerevisiae ESCRT-II (Vps22, Vps25, Vps36) and ESCRT-III subunits Vps20, Snf7, Vps2 are a gift from James Hurley (addgene plasmids #17633, #21490, #21492, #21494)(Wollert et al., 2009; Wollert and Hurley, 2010). The yeast Vps24 gene is subcloned into a pRSET(A) expression vector, containing a His6-tag and Enterokinase protease cleavage site. All constructs are transformed into JM109 (DE3). All proteins are expressed in autoinduction media, including trace elements (Formedium). 2 L of culture are grown in the presence of ampicillin (final concentration of 100 mg/L) for 3 hr at 37 °C and then the temperature is reduced to 25 °C overnight. Cells are broken with a cell disrupter (Constant systems), cooled to 4 °C and the supernatant is separated from the debris by centrifugation at 30,000 x g. ESCRT-II is purified as a complex (Vps22 contains a His_6 tag) using a HisPrep FF 16/10 (GE Healthcare) in PBS, with an elution gradient of imidazole up to 1 M. The eluted complex is buffer exchanged into PBS, flash frozen and stored −80° C until use. ESCRT-III proteins (Vps20, Snf7, Vps2) are affinity purified using MBP resin (GE Healthcare) and eluted with 10 mM maltose. Vps24 is denatured in 5 M urea and applied to IMAC Sepharose resin charged with Ni^{2+} (GE Healthcare) and after washing is eluted over a 0 - 500 mM imidazole gradient. Elution fractions (Vps20, Snf7, Vps2) are cleaved using TEV protease (pET28-MBP-TEV is a gift from Zita Balklava & Thomas Wassmer; addgene plasmid # 69929) (Currinn et al., 2016). TEV protease is removed with a second round of Ni^{2+} affinity chromatography. Pooled fractions are finally purified by size exclusion chromatography (SEC). The monomeric state of all proteins is verified using analytical SEC (Superdex 75 10/30 column; GE Healthcare) (**Figure S1 (i)**). Purified proteins are maintained

at concentrations not higher than 8 µM to avoid aggregation and aliquoted in small volumes (~50 µL) for one-time use only to avoid damaging from freeze-thawing. Aliquots are immediately flash frozen in liquid nitrogen and stored at −80° C until use. Full-length yeast Vps4 (a gift from James Hurley; addgene plasmid #21495) is purified from E. coli as a glutathione S-transferase fusion protein following the method in Wollert and Hurley 2009 (Wollert et al., 2009). The purified protein is concentrated to 20 µM, tested for ATPase activity and stored at −80 °C. Proteins are defrosted on ice prior to use.

Electroformation of Giant Unilamellar Vesicles (GUVs)

Preparation of GUVs is performed using the electroformation method. A solution of lipids in chloroform (15 µL, 0.7 mM; (1-palmitoyl-2-oleoyl-*sn*-glycero-3-phosphocholine (POPC, 61.9 mol%), 1-palmitoyl-2-oleoyl-*sn*-glycero-3-phospho-L-serine (POPS, 10 mol%), cholesterol (25 mol%), 1,2-dioleoyl-*sn*-glycero-3-phospho-(1'-myo-inositol-3'-phosphate) (PI(3)P, 3 mol%), and lissamine-rhodamine-PE (0.1 mol%), all purchased from Avanti Polar Lipids Inc. Alabaster, Al. USA.) is applied as a thin layer over two Indium-tin oxide (ITO) glass slides (8-12 Ω/sq, Sigma-Aldrich) and dried under a stream of nitrogen gas. The slides are assembled into an electroformation chamber using a silicone rubber gasket to create a chamber between the opposing conductive surfaces, with strips of copper tape between the gasket and each conductive surface to serve as electrical contacts. The chamber is filled with sucrose solution (600 mM) and sealed with a silicone rubber plug. The chamber is placed in an oven at 60 °C and using a function generator, an AC voltage was applied to the system (3 V peak to peak, 10 Hz, sinusoidal waveform) for two hours, followed by gradual reduction in the frequency over approximately 10 minutes to facilitate the closure of nascent GUVs from

the surface. The chamber is then allowed to cool to room temperature before the GUV suspension is harvested.

If desired, osmotic relaxation of membrane tension is achieved by overnight incubation at 4 °C of the resulting GUV suspension in Tris buffer (50 mM Tris, pH 7.4) adjusted to 10 mOsM higher than the sucrose electroformation solution (1:4, vol:vol Tris:GUV suspension) to facilitate the osmotic transport of water from the GUV lumens. Osmolarity is measured using an Advanced Instruments 3320 osmometer and adjusted by addition of NaCl.

Confocal microscopy - ILV counting

Confocal microscopy is performed on a Zeiss LSM 880 inverted system, using a Plan-Apochromat 40x/1.4 Oil DIC M27 objective lens, NA = 1.4. 8-well glass bottomed imaging chambers (ibidi GmbH) are prepared by passivation of the interior glass surface by incubation overnight in 5 % Bovine serum albumin (BSA) solution, followed by copious rinsing with MilliQ water. A GUV suspension is diluted 1:4 with osmotically balanced Tris buffer, unless previously diluted with 10 % hyperosmotic Tris buffer as described previously for membrane relaxation. To ensure efficient mixing for each experiment, solutions are combined in an Eppendorf tube, followed by gentle agitation of the tube and transfer of the solution to the imaging chambers. Proteins are thawed over ice, before being added to the tubes such as to give final concentration of 10 nM ESCRT-II, Vps20, Vps24 and Vps2, and 50 nM Snf7, unless otherwise stated. Aliquots of proteins are used only once and never re-frozen and thawed again. Fluorescently labelled dextran (M_r ~ 5,000 Da, Cascade blue labelled) is then added, followed by 200 µL of GUV suspension. Imaging is then performed after 20 minutes of incubation. Once imaging is complete, a second fluorescent dextran (M_r

~ 10,000 Da, AlexaFluor 488 labelled) is added to each sample, followed by 10 nM Vps4 and 1 µM ATP.$MgCl_2$ and further imaging is performed after a 20 minute incubation period. For the ILV counting experiments, a tile scanning technique with a 3.1 µm section thickness is employed to capture a cross sectional volume through a large number of GUVs. Only ILVs visibly containing encapsulated dextran dye are counted, indicating that they have formed after the addition of the proteins. The apparent diameters of observed ILVs was measured manually during counting and binned into categories of ≤0.5 µm, 1 µm, 2 µm, 3 µm etc. The counts associated with each of these categories were used to produce ILV size histograms (see **Supplementary Information Figures S5** and **S6**) and determine average ILV diameter values.

Flicker spectroscopy

GUV membrane tension is quantified by flicker spectroscopy (Alex Rautu et al., 2017; Bassereau et al., 2014; Yoon et al., 2010). This technique analyses the power spectrum of membrane undulations (mean square undulation amplitude $\langle |u(q)|^2 \rangle$ versus wavenumber q from image analysis of a confocal microscopy time series taken at the midpoint (equator) of the GUV. 1000 frames are taken per GUV at 1024x1024 pixel resolution, frame rate and pixel size varied on a case by case basis as the maximum magnification that could accommodate the whole GUV is used to maximize scan speed and these factors are accounted for in the MATLAB analysis software. Fitting $\langle |u(q)|^2 \rangle$ with the equation:

Equation 1. $$\langle |u(q)|^2 \rangle = \frac{k_B T}{2\sigma}\left[\frac{1}{q} - \frac{1}{\sqrt{\frac{\sigma}{\kappa} + q^2}}\right]$$

allows us to quantify the bending rigidity (κ) and tension (σ) of individual GUV membranes (Pécréaux et al., 2004). The MATLAB program for GUV flicker analysis was kindly provided to

us by Prof. Pietro Cicuta and co-workers at the University of Cambridge, UK. Example flicker spectra are shown in supplementary **Figure S2** and **S3**.

Supplemental references

Alex Rautu, S., Orsi, D., Michele, L.D., Rowlands, G., Cicuta, P., S. Turner, M., 2017. The role of optical projection in the analysis of membrane fluctuations. Soft Matter 13, 3480–3483. https://doi.org/10.1039/C7SM00108H

Bassereau, P., Sorre, B., Lévy, A., 2014. Bending lipid membranes: Experiments after W. Helfrich's model. Adv. Colloid Interface Sci., Special issue in honour of Wolfgang Helfrich 208, 47–57. https://doi.org/10.1016/j.cis.2014.02.002

Currinn, H., Guscott, B., Balklava, Z., Rothnie, A., Wassmer, T., 2016. APP controls the formation of PI(3,5)P2 vesicles through its binding of the PIKfyve complex. Cell. Mol. Life Sci. 73, 393–408. https://doi.org/10.1007/s00018-015-1993-0

Pécréaux, J., Döbereiner, H.-G., Prost, J., Joanny, J.-F., Bassereau, P., 2004. Refined contour analysis of giant unilamellar vesicles. Eur. Phys. J. E 13, 277–290. https://doi.org/10.1140/epje/i2004-10001-9

Wollert, T., Hurley, J.H., 2010. Molecular mechanism of multivesicular body biogenesis by ESCRT complexes. Nature 464, 864–869. https://doi.org/10.1038/nature08849

Wollert, T., Wunder, C., Lippincott-Schwartz, J., Hurley, J.H., 2009. Membrane scission by the ESCRT-III complex. Nature 458, 172–177. https://doi.org/10.1038/nature07836

Yoon, Y.Z., Hale, J.P., Petrov, P.G., Cicuta, P., 2010. Mechanical properties of ternary lipid membranes near a liquid–liquid phase separation boundary. J. Phys. Condens. Matter 22, 062101. https://doi.org/10.1088/0953-8984/22/6/062101
